# The AKI-to-CKD Transition: The Role of Uremic Toxins

**DOI:** 10.3390/ijms242216152

**Published:** 2023-11-10

**Authors:** Camille André, Sandra Bodeau, Saïd Kamel, Youssef Bennis, Pauline Caillard

**Affiliations:** 1 Department of Clinical Pharmacology, Amiens Medical Center, 80000 Amiens, France; bodeau.sandra@chu-amiens.fr (S.B.); bennis.youssef@chu-amiens.fr (Y.B.); 2 GRAP Laboratory, INSERM UMR 1247, University of Picardy Jules Verne, 80000 Amiens, France; 3 MP3CV Laboratory, UR UPJV 7517, University of Picardy Jules Verne, 80000 Amiens, France; kamel.said@chu-amiens.fr (S.K.); caillard.pauline@chu-amiens.fr (P.C.); 4 Department of Clinical Biochemistry, Amiens Medical Center, 80000 Amiens, France; 5 Department of Nephrology, Dialysis and Transplantation, Amiens Medical Center, 80000 Amiens, France

**Keywords:** acute kidney injury, chronic kidney disease, AKI-to-CKD transition, uremic toxins

## Abstract

After acute kidney injury (AKI), renal function continues to deteriorate in some patients. In a pro-inflammatory and profibrotic environment, the proximal tubules are subject to maladaptive repair. In the AKI-to-CKD transition, impaired recovery from AKI reduces tubular and glomerular filtration and leads to chronic kidney disease (CKD). Reduced kidney secretion capacity is characterized by the plasma accumulation of biologically active molecules, referred to as uremic toxins (UTs). These toxins have a role in the development of neurological, cardiovascular, bone, and renal complications of CKD. However, UTs might also cause CKD as well as be the consequence. Recent studies have shown that these molecules accumulate early in AKI and contribute to the establishment of this pro-inflammatory and profibrotic environment in the kidney. The objective of the present work was to review the mechanisms of UT toxicity that potentially contribute to the AKI-to-CKD transition in each renal compartment.

## 1. Introduction

Acute kidney injury (AKI) and chronic kidney disease (CKD) are increasingly more frequent and are therefore becoming major public health concerns. In a recent study, the incidence rate for AKI and acute kidney disease (AKD) (standardized by age and sex) ranged from 134 to 162 events/10,000 person-years [1]. CKD affects around one person in ten [2] and mostly results from two cardiovascular disease risk factors: arterial hypertension and diabetes. CKD and AKI are interconnected, and each is a risk factor for the other [3,4]. Patients who experience an episode of AKI—even a nonserious one—have a ~25% greater risk of progression to CKD [5]. Both CKD and AKI are associated with higher morbidity and mortality rates in general and cardiovascular morbidity and mortality rates in particular [6,7,8]. CKD is known to accelerate the arterial and valvular calcification process and promote cardiovascular events, such as acute coronary syndrome and stroke [6,9]. A number of recent studies have demonstrated the link between AKI, cardiovascular events, and mortality independent of CKD [10,11]. Patients are more likely to develop acute heart failure and hypertension in the years following an episode of AKI [8,10,11,12,13]. Given that both CKD and AKI are associated with a higher mortality rate, it is important to limit their occurrence and, notably, to intervene early with regard to the AKI-to-CKD transition.

**Figure 1:**
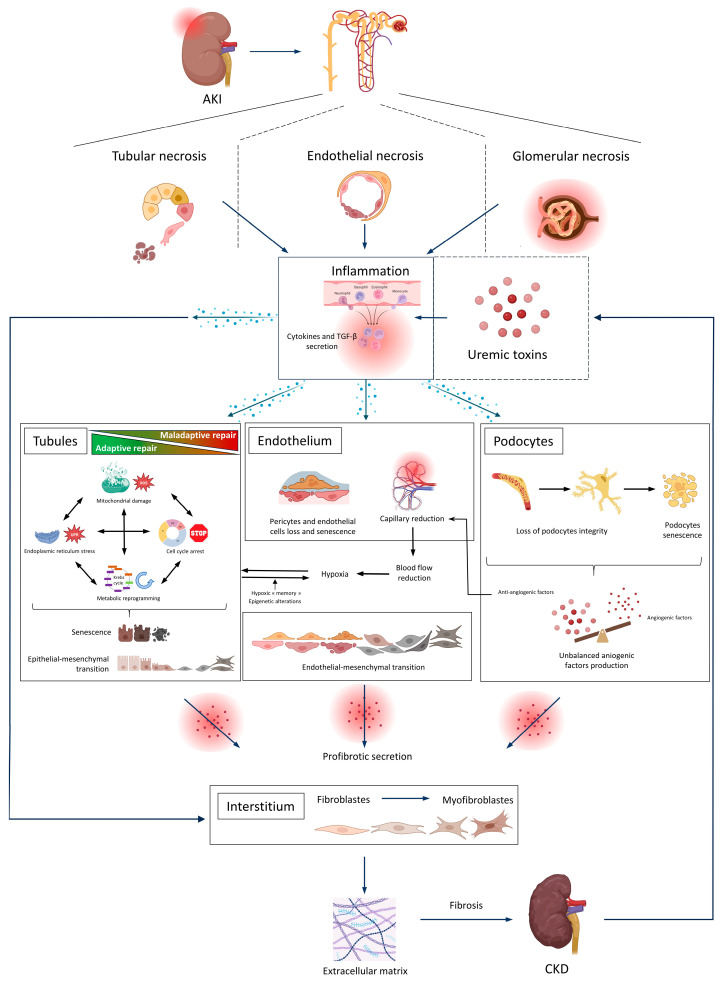
Tubular, endothelial, and glomerular mechanisms leading to the AKI-to-CKD transition and the involvement of UTs. After tubular, endothelial, or glomerular injury, necrosed cells stimulate the innate system and then the adaptive immune system. Imbalanced cytokine and TGF-β secretion leads to tubular maladaptive repair (mitochondrial dysfunction, ER stress, cell cycle arrest, metabolic reprogramming, tubular senescence, and EMT), endothelial senescence and rarefaction, hypoxia, and podocyte loss. Across several compartments, the overproduction of profibrotic factors stimulates extracellular matrix-secreting myofibroblasts, increases fibrosis, and hastens progression to CKD. AKI, acute kidney disease; CKD, chronic kidney disease; EMT, epithelial-to-mesenchymal transition; ER, endoplasmic reticulum. Created with BioRender.com.

With a view to better understanding the risk of CKD progression after AKI and to identify potential new therapeutic targets, several research groups have explored the underlying cellular and molecular mechanisms [14]. These mechanisms involve all the compartments of the kidney (i.e., tubular, interstitial, vascular, and glomerular components) and a variety of pathways [14]. It is also thought that various patient-related and AKI-related factors (including uremic toxins (UTs)) influence the AKI-to-CKD transition [15,16]. Indeed, UTs (particularly indoxyl sulfate (IS) and para-cresyl sulfate (pCS)) are known to accumulate in the serum during CKD and AKI and are associated with CKD-related elevation in cardiovascular risk [17,18,19]. The objective of the present study was to review (i) the mechanisms involved in the AKI-to-CKD transition and (ii) UTs’ potential role in this transition. Figure 1 provides a schematic view of the potential impact of UTs on the AKI-to-CKD transition.

## 2. Acute and Chronic Kidney Diseases and Uremic Toxins: Definitions

### 2.1. AKI as a Risk Factor for CKD

#### 2.1.1. Definition and Epidemiology of AKI

AKI is defined as a sudden loss of kidney function, as characterized by an elevation in the serum creatinine level and/or a decrease in urine output. These two criteria are used to classify AKI as being mild, moderate, or severe, according to the Kidney Disease Improving Global Outcomes classification [20]. The incidence of AKI is increasing, and the condition is frequently encountered in hospitals in general and in intensive care units (ICUs) in particular [21,22]. More than 50% of ICU patients experience an episode of AKI [23,24]. AKI is associated with a high mortality rate; this can be as high as 50% in patients who require dialysis [23,25]. There are several risk factors for AKI, including patient-related factors, such as older age, pre-existing chronic organ failure (especially kidney, heart, or liver failure), cancer or auto-immune disease, and AKI etiology [26,27,28,29]. Pre-existing CKD is the most significant risk factor for AKI; it increases the risk more than 10-fold [30].

Except in certain specific cases of glomerulus injury, the main histological findings of AKI are acute tubular lesions and interstitial infiltrates. The tubule is very sensitive to injury by ischemia (induced mainly by septic or cardiogenic shock) and drug toxicity [31,32,33]. The pathogenesis of acute tubular injury is a step-wise process [34,35]. The first phase of acute tubular injury is characterized by tubular epithelial cell damage, tubular luminal dilatation, proximal loss of the brush border, and loss of nuclei. Next, ischemic damage, endothelial and tubular epithelial cell necrosis, and apoptosis are observed. Tubular cell detachment can also obstruct the tubular lumen. In the third step, the release of pro-inflammatory molecules worsens the existing damage. The last steps are marked by cell repair, cell regeneration, and the reestablishment of normal tubular function; this phase has a central role in the transition from AKI to recovery or CKD.

#### 2.1.2. Changes in Kidney Function after an Episode of AKI

AKI is also a risk factor for CKD, and there is a continuum between the episode of AKI and the development of CKD. In addition to its severity, AKI is defined by its duration (less than 7 days) [36] and CKD by a persistent loss of kidney function over 3 months. Acute kidney disease corresponds to the AKI-to-CKD transition, i.e., between 7 days and 3 months post-onset. Histological analyses show that CKD damages all four compartments of the kidney [37,38]. Interstitial fibrosis is the most frequent early-onset histological change and is correlated with the progression of CKD [39]. Tubular atrophy is the second most frequent change observed in CKD and is also associated with the degree of renal impairment [40]. Several cellular mechanisms (myofibroblast activation, a tubular epithelial-to-mesenchymal transition (EMT), inflammatory molecule production, immune cell recruitment, and matrix expansion and remodeling) and pathways (Notch and Wnt) have been considered fibrosis markers and targets for slowing down the progression of CKD [41].

Various patient-related and AKI-related risk factors are associated with the course of CKD [15,16]. Thus, gender (men), older age, comorbidities (such as uncontrolled diabetes and hypertension and cardiovascular disease), sociodemographic disparities and genetic factors contribute to the development and progression of CKD after AKI [42,43,44,45,46,47]. Factors linked to AKI (the severity, etiology, and number of episodes) all contribute to the AKI-to-CKD progression [48,49,50]. Thus, the risk of end-stage renal disease increases with AKI severity; in a meta-analysis encompassing 2,017,437 patients, the hazard ratio for CKD was 2.7 in AKI stage 1, 3.4 in AKI stage 2, and 8.9 in AKI stage 3 [51,52,53]. Moreover, each episode of AKI doubles the likelihood of progression to CKD after AKI [54].

It is noteworthy that many of the risk factors for the AKI-to-CKD transition are also risk factors for AKI onset [55]. Based on those findings in general and histological kidney lesions observed during AKI in particular, some researchers have looked for potential biomarkers of CKD risk. In a longitudinal study of 656 patients, increases in the urine concentrations of kidney injury molecule-1 (KIM-1), monocyte chemoattractant protein-1 (MCP-1), and plasma tumor necrosis factor receptor (TNFR)-1 during the 12 months after AKI were associated with a two- to three-fold greater risk of developing CKD [56]. Furthermore, preclinical and clinical studies suggest that neutrophil gelatinase-associated lipocalin (NGAL) is a marker of non-recovery from AKI, whereas urine uromodulin is associated with a reduction in CKD risk [56,57]. The accumulation of metabolites (and especially uremic toxins) might also have a role in the AKI-to-CKD transition.

### 2.2. Uremic Toxins

**Figure 2:**
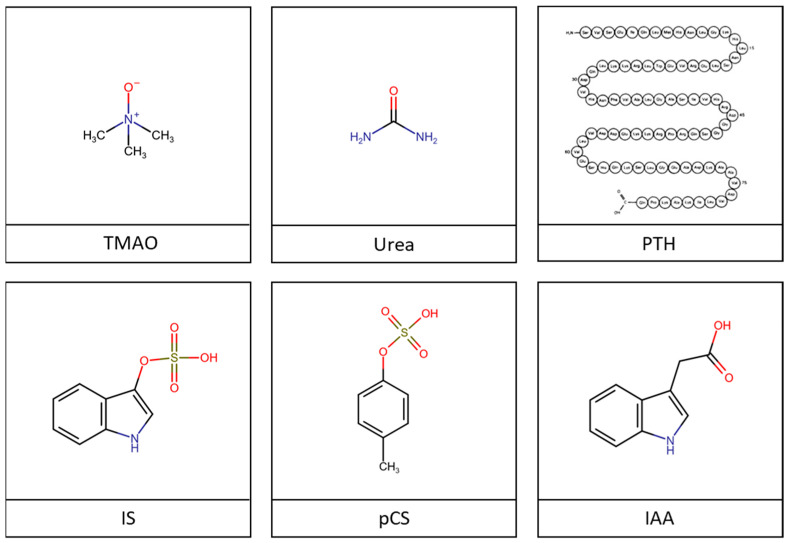
Chemical structure of UTs. TMAO and urea belong to the group of water-soluble UTs with low molecular weight (MW < 500 Da). PTH belongs to the group of water-soluble UTs with high molecular weight (MW > 500 Da). IS, pCS, and IAA belong to the group of protein-binding UTs. IAA, indole-3-acetic acid; IS, indoxyl sulfate; pCS, para-cresyl sulfate; PTH, parathyroid hormone; TMAO, trimethylamine-N-oxide.

During CKD, the kidneys’ reduced filtration capacity results in the retention of numerous metabolites called uremic toxins (UTs). A UT is defined as a molecule that accumulates in the blood and organs of CKD patients, where it exerts deleterious effects on biological functions [58]. According to this definition, some 150 molecules are considered to be UTs and are classified by the EUTOX (European Uremic Toxins Work Group) into three groups depending on their physico-chemical properties [59,60]: water-soluble UTs (molecular weight (MW) < 500 Dalton) with urea and trimethylamine-N-oxide (TMAO) (Figure 2); water-soluble UTs (MW > 500 Dalton), including peptides and small proteins (β2-microglobulin or parathyroid hormone); and protein-bound UTs (PBUTs), which are strongly bound to plasma proteins, preventing them from renal filtration elimination and therefore from hemodialysis, including indoxyl sulfate (IS), para-cresyl sulfate (pCS), and indole-3-acetic acid (IAA).

UT precursor compounds are derived from the metabolism of certain dietary amino acids by the gut microbiota (GM). They pass the intestinal barrier and reach the liver through portal circulation, where they are metabolized by hepatic enzymes (sulfotransferase family 1A member 1 (SULT1A1) and flavin-containing mono-oxygenase 3 (FMO3)) into UTs. Once the UTs reach the systemic compartment, protein-binding UTs (including IS and pCS) are strongly bound to plasma proteins (mainly on albumin’s Sudlow sites) [61]. Lastly, UTs are secreted by the kidney after being taken up by the basolateral organic anion transporters 1/3 (OAT_1_/OAT_3_) expressed by proximal tubular cells [62,63].

Protein-bound UT (PBUTs) accumulation was first described in CKD patients. Indeed, in CKD patients, plasma PBUT concentrations increase from 27 times for IAA to 68 times for IS compared to healthy patients [64]. The clinical symptoms caused by UT toxicity are referred to collectively as “uremic syndrome.” Several preclinical studies describe an impairment of cardiac, neuron, and kidney cell damage involving the activation of the aryl hydrocarbon receptor (AhR) and nuclear factor-kappa B (NF-κB), reactive oxygen species (ROS) production, and the mitogen-activated protein kinase (MAPK), extracellular signal-regulated kinase (ERK), and transforming growth factor β (TGF-β) signaling pathways in endothelial cells (ECs) [65,66], astrocytes [67], the blood–brain barrier [68], and podocytes [69]. This newly acquired pro-inflammatory phenotype leads to fibrosis and increased apoptosis, together with renal dysfunction [70], ventricular dysfunction [71], and a poor cognitive state [72,73]. Several clinical studies in CKD patients have shown that UTs promote cardiovascular morbimortality [18,74,75], poor post-stroke recovery [76,77], cognitive impairment [78], and a greater risk of fractures [79].

Plasma UT concentrations in AKI are lower than plasma UT concentrations in CKD [17,80,81]. However, studies of the accumulation kinetics of these UTs in AKI showed contradictory data. In contrast to CKD, some UTs do not accumulate (p-cresyl glucuronide (pCG) and 3-carboxy-4-methyl-5-propyl-2-furanpropionate (CMPF)) [17,80,82]. In vitro and ex vivo studies have already observed UTs’ (IS, pCS, p-cresol) deleterious effects (oxidative stress, apoptosis, wound repair) after short exposure (30 min–1 h) in human umbilical vein endothelial cells (HUVECs) and vascular smooth muscle cells (VSMCs) [83,84,85]. Although several clinical studies shift towards a link between transient accumulation of PBUTs and cardiovascular complications during AKI [86], the role of PBUTs in the progression of renal damage remains to be determined, and several mechanisms directly implicate these molecules in the AKI-to-CKD transition.

## 3. Uremic Toxins in the AKI-to-CKD Transition

### 3.1. Gut Microbiota Dysbiosis and AKI-to-CKD Transition

The GM plays a central role in the genesis of UTs, is directly linked to AKI and CKD [87], and is located at the center of the bidirectional intestine–kidney axis [88,89]. Both AKI and CKD are responsible for alterations in the GM. In the case of CKD, several factors (a low fiber intake or specific treatment with phosphate binders and iron supplements) are responsible for this dysbiosis favoring the accumulation of UTs [90,91]. In addition to these factors, AKI is specifically associated with reduced levels of short-chain fatty acids produced by commensal bacteria in the microbiota [92]. This phenomenon is linked to systemic inflammation, which also promotes the formation of UTs [93,94].

On the other hand, GM dysbiosis is also associated with the progression of renal disease. Under physiological conditions, the microbiota functions with a low O_2_ supply [95]. However, GM dysbiosis alters these hypoxic conditions, leading to inflammation and apoptosis and contributing to the AKI-to-CKD transition [96]. GM dysbiosis also influences mitochondrial dysfunction, leading to ROS increase and AhR signaling through UT synthesis (IS, pCS, TMAO) and therefore to the progression of CKD [97,98,99].

### 3.2. The Tubulo-Interstitial Compartment

#### 3.2.1. Maladaptive Repair and the EMT

##### In the AKI-to-CKD Transition

After injury, tubular repair allows the patient to recover renal function and is achieved through (i) the regeneration of the lost tubular epithelial cells by dedifferentiation–proliferation–redifferentiation of surviving tubular epithelial cells and (ii) temporary, circumscribed, renal fibrosis as a short-term, adaptive tissue repair mechanism. However, the persistence of fibrosis after severe, repetitive injury leads to maladaptive repair, with replacement of the renal parenchyma by connective tissue, preventing the total recovery of the estimated glomerular filtration rate (eGFR) present before the AKI.

Persistent fibrosis is maintained by cells having undergone partial EMT, although the origin of these cells is still subject to debate [100]. EMT is a pathological mechanism in which tubular epithelial cells transdifferentiate into a fibroblast-like phenotype during tubulo-interstitial dialogue. The loss of epithelial cell junctions and polarity is followed by a change in protein expression (under-expression of the epithelial proteins zonula occludens-1 (ZO-1) and E-cadherin) and over-expression of mesenchymal markers (e.g., alpha-smooth muscle actin (α-SMA), vimentin, and fibronectin). This leads to the acquisition of a pathological extra-cellular matrix (ECM) secretory phenotype and to migratory capacity [101,102,103]. Maladaptive transdifferentiation sustains persistent, uncontrolled fibrosis, which drives the AKI-to-CKD transition through several phenotypic changes: cell cycle arrest, reactivation of proliferation pathways, organelle stress, and epigenetic alterations.

##### The Involvement of UTs

The results of several preclinical studies have shown that UTs accumulate in tubular cells [104] and contribute to EMT by promoting epithelial marker loss and mesenchymal marker acquisition via induction through TGF-β (Table 1). Exposure of proximal renal tubular NRK-52E cells to IS alters the typical cubic aspect of epithelial cells and turns them into fibroblast-like cells. The epithelial phenotype (ZO-1 and E-cadherin) is replaced by a mesenchymal phenotype (with overexpression of α-SMA) associated with induction of the ERK1/2 and MAPK apoptotic pathways. Pretreatment with the OAT_3_ inhibitor probenecid and inhibitors of the ERK1/2 and MAPK pathways reduced IS-induced EMT [105]. Similarly, exposure of murine proximal tubule cells and an in vivo mouse model to the UTs IS and pCS activated TGF-β excretion and initiated EMT via the Smad and Snail pathways [106,107]. Fibroblasts are also activated in mice exposed per os to IS [108]. Administration of AST-120 (Kremezin^®^) to mice allows epithelial cells to undergo EMT [109,110,111]. Senescence and endoplasmic reticulum (ER) stress promoted by IS are cellular adaptations for progression to a mesenchymal phenotype and are attenuated by the administration of a chelating resin [111]. To date, the influence of UTs on the expression of EMT markers has not been investigated in patients with renal disease.

#### 3.2.2. Hypoxia

##### In the AKI-to-CKD Transition

During AKI, an initial acute injury may expose tubular cells to hypoxia. The kidneys are particularly sensitive to hypoxia [147,148,149]. Under hypoxic conditions, stimulation of oxidative stress, an imbalance between the mitochondrial production and the elimination of ROS (principally the peroxide ion O_2_^2−^ and the superoxide ion O_2_^•−^), activates the hypoxia-inducible factor (HIF) heterodimer, which enables the transcription of genes that compensate for oxygen scarcity (angiogenesis, erythropoiesis, anaerobic glycolysis) [150,151]. Hypoxia contributes to the AKI-to-CKD transition by promoting a mesenchymal phenotype via tubulointerstitial fibrosis [152,153]. The involvement of TGF-β and tumor protein 53 (p53) as intermediates between ROS secretion and tubular injury has also been demonstrated [154]. Hypoxia-triggered EMT can be inhibited using HIF siRNA [155]. In a mouse model of ischemia/reperfusion (I/R), exposure to a molecule mimicking the action of the superoxide dismutase (SOD) or the nuclear factor erythroid 2-related factor 2 (Nrf2) reduced ROS and peroxidized lipid production, apoptosis, renal tubular damage, and the number of CD68+ cells, promoting macrophage infiltration [156,157].

##### The Involvement of UTs

The literature data show that UTs are capable of inducing hypoxia and ROS secretion—both of which are involved in the AKI-to-CKD transition [158,159]. Nakagawa et al. showed that exposure of human proximal tubular (HK2) cells to IS and IAA led to intracellular ROS production [120]. This effect was suppressed by probenecid (OAT_3_ inhibitor) and antioxidants (NAC or probucol) [112]. Shimizu et al. demonstrated that ROS activation by IS is achieved through NF-κB. Exposure to NF-κB inhibitors (siRNA, pyrrolidine dithiocarbamate, and isohelenin), an antioxidant (NAC), and an NADPH oxidase inhibitor (diphenyleneiodonium (DPI)) reduces the effect of IS on angiotensinogen production by tubular cells and lessens fibrosis and inflammation [118]. Bolati et al. confirmed the involvement of oxidative stress in an in vivo model; the inhibition of transcription factor Nrf2 by IS via NF-κB led to a decrease in levels of the antioxidant enzymes heme oxygenase 1 (HO-1) and NAD(P)H quinone dehydrogenase 1 (NQO1) [119]. Furthermore, the antioxidant action of HO-1 and NQO1 was restored by administration of AST-120 to the animals. Animals exposed per os to indole (an IS precursor) developed kidney hypoxia and damage at 16 weeks [121]. Administration of AST-120 restored physiological oxygen consumption and reduced hypoxia and kidney damage. An improvement was also observed in vitro with the use of the Na/K/ATPase inhibitor ouabain and the NADPH oxidase inhibitor apocynin. Lastly, in a cohort of CKD patients, a negative correlation was identified between plasma concentrations of the UTs N-methyl-2-pyridone-5-carboxamide and N-methyl-4-pyridone-5-carboxamide (2PY and 4PY) and the plasma concentration of reduced glutathione (GSH), the most abundant antioxidant and a major detoxification agent in cells [142]. Inhibition of this protective molecule increases the risk of oxidative cells damages and can lead to CKD [160].

#### 3.2.3. Organelle Stress

##### In the AKI-to-CKD Transition

The mitochondria is the powerhouse of the cell, and ROS-dependent mitochondrial stress is associated with many kidney diseases [161]—including AKI and the AKI-to-CKD transition [162]. Several in vivo studies have confirmed the involvement of mitochondrial stress in renal tubules in the progression from AKI to CKD through various mechanisms: redox alterations (ROS production, decreased β-oxidation, depolarization of membrane potential) [163], activation of mitochondrial fission [164], and decreased mitochondrial ATP synthesis [165]. Clinical studies have corroborated these data, as shown by Hu et al.’s report of a correlation between the urine mitochondrial DNA concentration and markers in patients with severe AKI relative to patients with mild AKI [166].

Permanent triggering of the unfolded protein response (UPR) pathway during ER stress activates apoptotic pathways via the CEBP-homologous protein (CHOP, a molecular intermediate triggered by ER stress and a mediator of apoptosis) and promotes the AKI-to-CKD transition [167,168,169]. Lastly, clinical studies have corroborated the preclinical results by highlighting a correlation between ER stress marker activation (CHOP, X-box binding protein 1, and ER-associated protein reticulon 1A) and a decrease in the eGFR [170]. These markers were also overexpressed in AKI patients with progression relative to AKI patients without progression [171].

##### The Involvement of UTs

The AKI-to-CKD transition might also involve stressed organelles, including mitochondria. Some UTs (notably, hippuric acid (HA), IAA, and uric acid) can be produced by mitochondria via physiological or pathological pathways [172]. UTs (such as IS, pCS, HA, and CMPF) may be responsible for mitochondrial stress in renal tubular cells, as has already been shown in muscle [173,174] and the endothelium [84]. Mutsaers et al. showed that exposure of CiPTEC kidney cells to UTs inhibited mitochondrial succinate dehydrogenase (complex II) activity by over 20% compared with controls. There was also an 18% reduction in the capacity of the electron transport system [122]. Aerobic and anaerobic metabolic pathways were impaired in a model of nephrectomized B-6 mice treated with IS and pCS; the increased mitochondrial fission and autophagy were similar to those observed in senescent cells and in AKI [123]. Lastly, a study of a cohort of hemodialysis patients showed that CMPF reduced mitochondrial respiration by inhibiting ADP-dependent oxidation of NADH-dependent substrates [143].

A few studies have investigated the induction of ER stress by UTs in kidney. For example, Jeon et al. found that apigenin treatment in an HK-2 cell model attenuated the ER stress induced by IS and led to a decrease in interleukin (IL)-6 and cyclin-dependent kinase inhibitor 1 (p21) levels [124]. Similar results were found in vivo. Indeed, administration of AST-120 in a nephrectomized rat model reduced the plasma concentrations of IS and CHOP. Inhibition of activating transcription factor-(ATF)-4 (the endoplasmic receptor activating the UPR pathway) by siRNA reduced the expression of IL-6, a proinflammatory cytokine involved in fibrosis [125].

#### 3.2.4. Metabolic Reprogramming

##### In the AKI-to-CKD Transition

Metabolic reprogramming is the ability of cells to adapt their metabolic response to changes in their environment. Tubulointerstitial cells need a lot of ATP to carry out their excretion and reabsorption functions. These functions depend mainly on the β-oxidation of fatty acids, since glucose is reabsorbed by sodium glucose cotransporters (SGLTs), making glycolysis less available. During AKI, β-oxidation decreases and ATP synthesis is compensated through aerobic glycolysis (insufficient to meet NADH and FADH_2_ cofactor requirements), followed by anaerobic glycolysis (leading to lactate synthesis). Activation of the HIF-1α factor and activation of AhR [175] and mitochondrial dysfunction [176] are both causes and consequences of the transition from normoxic to anaerobic metabolism. In addition, the processes involved in the progression of renal pathology (collagen deposition, chronic inflammation, etc.) require large amounts of energy [177,178,179].

##### The Involvement of UTs

Some UTs arise directly from metabolic reprogramming, notably, kynurenine [177] and lipid intermediates [180]. However, they are as much the result of kidney damage as the driving force behind its progression. Hopp et al. showed a link between kidney damage, metabolic reprogramming, and the accumulation of UTs, where an alteration in tryptophan metabolism was associated with a deterioration in kidney function and the accumulation of UTs in an in vivo model of autosomal dominant polycystic kidney disease [127]. UTs also stimulate inflammation via an AhR-dependent reprogramming of arachidonic acid metabolism [126].

#### 3.2.5. Epigenetic Alterations and Cell Cycle Arrest

##### In the AKI-to-CKD Transition

Epigenetic alterations triggered by hypoxia via the HIF (histone modifications, DNA methylation, non-coding RNAs, and chromatin conformational changes) form the pathophysiological “hypoxic memory” of the primary injury [181]. These epigenetic changes are conserved in kidney cells after AKI and predispose the cell to progression to CKD [182]. Several in vivo studies have shown that epigenetic alterations aggravate AKI [183,184,185,186]. A number of clinical studies have confirmed these data: methylation-enriched areas in renal biopsies [187], hypermethylation of the kallikrein gene [188], and overexpression of circulating RNAs [189,190].

In response to severe or repeated injury, tubular cells exhibit G2/M cell cycle arrest (as opposed to G1/0 arrest under normal conditions); this leads to premature renal tubular cell senescence in AKI [191] and CKD [192]. Several in vitro models have evidenced a link between G2/M phase arrest-triggering senescence (p21) and transdifferentiation towards a pro-inflammatory phenotype (α-SMA, fibronectins, and fibroblast size) [193,194]. In vivo studies have confirmed these data [195,196]. Furthermore, NAC reduced cisplatin-induced renal senescence in a C57BL/6 mouse model [197]. Several clinical studies have found that two urine biomarkers of cell cycle arrest (tissue inhibitor metalloproteinase (TIMP)-2 and insulin-like growth factor-binding protein (IGFBP)-7) predict the imminent risk of AKI in critical care patients [198].

##### The Involvement of UTs

The results of a few preclinical studies have illustrated the effects of UTs on epigenetic alterations in renal tubular cells and thus their contribution to the AKI-to-CKD transition. In a study of HK-2 cells, for example, IS increased the methylation of secreted frizzled-related protein (sFRP) CpG islands (inhibiting its expression) and led to Wnt/β-catenin pathway activation and to aberrant kidney fibrosis. Administration of recombinant sFRP5 in vivo alleviated IS-induced fibrosis [128]. In a nephrectomized B-6 mouse model, exposure to IS and pCS induced expression of the DNA methytransferase involved in the hypermethylation of Klotho; this abrogated Klotho expression in renal tubules [129], as also seen in AKI [199]. The impact of UTs on renal cell cycle arrest initiates molecular pathways involved in the AKI-to-CKD transition. In a renal tubular cell model, D-serine (a novel UT) induced senescence via activation of general control nonderepressible 2 (GCN2) [145]. In the same manner, IS promoted senescence in HK-2 cells through activation of NF-κB, leading to TGF-β overexpression, p53 and p21 activation, and senescence. These effects were blocked by the NF-κB inhibitors pyrrolidine dithiocarbamate and isohelenin. Similar results were found in a rat CKD model, in which AST-120 administration decreased the upregulation of phosphorylated p65, p53, p21, β-galactosidase, TGF-β, and α-SMA [130,131,132].

#### 3.2.6. The TGF-β Signaling Pathway

##### In the AKI-to-CKD Transition

TGF-β is produced by dedifferentiated epithelial tubular cells after renal injury; it promotes epithelial dedifferentiation and cell cycle arrest, sensitizes cells to apoptosis, shortens peritubular capillaries, aggravates hypoxia, and favors progression from AKI to CKD. Furthermore, TGF-β stimulates EMT and aggravating fibrosis. Lastly, TGF-β acts as a chemoattractant for macrophages and promotes their infiltration of the tubules. Knock-out of the gene for TGF-β receptor II (TGF-βRII) in a mouse model attenuated the symptoms of AKI [200]. Furthermore, control mice showed greater TGF-β expression, fibrosis, and macrophage infiltration than TGF-βRII-/- mice [201,202], prevented by the use of genetic or pharmacologic TGF-β inhibitors [203]. Finally, a review of nephrodiabetic patients showed that TGF-β induced EMT in both the proximal and the distal region of the nephron [204].

##### The Involvement of UTs

Several studies have demonstrated that UTs stimulate the production of TGF-β, a central mediator in the progression of tubules towards fibrosis. A growing body of evidence from preclinical studies points in this direction. In an HK-2 cell model, for example, IS induced TGF-β expression and thus expression of the EMT marker α-SMA [113]. Another UT (IAA) is also capable of inducing TGF-β [112]. Several in vivo studies have demonstrated that administration to animals of asymmetric dimethylarginine (a water-soluble UT), IS or pCS is associated with oxidative stress, elevated TGF-β, EMT, and renal fibrosis [106,107,146]. Lastly, administration of AST-120 decreased UT and TGF-β levels and thus reduced renal fibrosis—confirming UTs’ involvement in renal EMT [114,115,116,117].

#### 3.2.7. Inflammation

##### In the AKI-to-CKD Transition

During the activation of the adaptive immune system, the balance between pro-inflammatory phenotypes (M1 macrophages and interferon gamma-secreting lymphocytes) and anti-inflammatory phenotypes (M2 macrophages and IL-10-secreting lymphocytes) favors (or not) tubular repair and fibroblast overactivation and thus determines the resolution of AKI or its progression to CKD. Several studies of preclinical models have shown that the hypersecretion of pro-inflammatory cytokines by macrophages is involved in the progression of AKI to CKD [205]. Mitochondrial injury can also induce inflammation [206,207]. The results of a number of clinical studies have confirmed these findings. Histological analysis of post-mortem renal biopsies from patients with AKI after septic shock showed intense leukocytic infiltration and marked apoptosis [208]. In a study of a cohort of post-surgical AKI patients, elevated C-reactive protein (CRP) levels were associated with higher mortality [209].

##### The Involvement of UTs

UTs stimulate the production of proinflammatory molecules by immune cells. This was demonstrated by Sun et al.’s study of inflammation genes expressed by renal tubular cells after exposure to IS and pCS. The cytokines IL-6, IL-15, and TGF-β were overexpressed, as were the NF-κB, Smad, Stat, and apoptosis pathway components (B2m, Bax, and Bcl2) [133]. This effect was attenuated by treatment with anti-Stat3 siRNA [132], indole propionic acid antioxidant [134], and AST-120 [135]. In a cohort of patients with abdominal aortic aneurysm (AAA) and matched control patients, there was a positive correlation between the plasma IS concentration and the proportion of proinflammatory CD14+/CD16+ monocytes. After exposure of THP1 monocytes to increasing concentrations of IS or serum from AAA patients, the cells differentiated into macrophages secreting proinflammatory cytokines (IL-6, MCP-1, and cyclooxygenase 2 (COX2)) [136]. The induction of MCP-1 expression by IS was also observed in vivo and was associated with renal monocyte/macrophage infiltration [137].

#### 3.2.8. Iron Death Pathways

##### In the AKI-to-CKD Transition

Ferroptosis is a type of cell death that is different than apoptosis and necrosis, playing a role in the AKI-to-CKD transition [210]. Iron metabolism is a key factor in initiating the ferroptosis process via the accumulation of products of lipid peroxidation and overproduction of ROS [211,212]. Several cellular pathways involved in the AKI-to-CKD transition depend on ferroptosis (mitochondrial dysfunction, fibrosis) [213,214,215]. Inflammation is closely linked to ferroptosis, and they favor each other. On the one hand, multiple metabolites produced during the inflammatory state increase the susceptibility of the kidney to ferroptosis [216,217], and on the other hand, ferroptosis is able to promote the production of damage-associated molecular patterns (DAMPs) and inflammatory mediators [218,219]. In a mouse model of AKI, pre-treatment with an anti-oxidant, FG-4592, reduces the progression of fibrosis by blocking ferroptosis [220]. Finally, ferroptosis is also interconnected with other forms of cell death (necrosis, pyroptosis, eryptosis), which accelerates the destruction of renal tubular cells [221,222]. Eryptosis (programmed erythrocytes death) is involved in anemia during CKD [223] and depends on iron homeostasis and oxidative stress [224,225].

##### The Involvement of UTs

Only a few studies have examined the role of UTs in ferroptosis during AKI-to-CKD transition. In a recent in vivo study, the authors observed a higher level of lipid peroxidation and a higher iron concentration in the kidneys of AKI mice, in association with overexpression of ferroptosis proteins (such as transferring receptor 1 (TfR1)) [144]. PBUTs have also been implicated in ferroptosis in vitro. CMPF showed the greatest effect, inhibiting the expression of ferritin and GPX4, a protein decreasing lipid peroxidation. In another study, the authors demonstrated the involvement of IS in iron metabolism during CKD [138]. Indeed, IS increases the gene and protein expression of hepcidin through AhR. Moreover, IS and IAA promote eryptosis via cytosolic Ca^2+^ and ROS accumulation, aggravating anemia and CKD [139,140,141,226].

### 3.3. Endothelial Dysfunction

#### 3.3.1. In the AKI-to-CKD Transition

Renal microcirculation in general and peritubular capillaries in particular are very susceptible to injury [14]. Vascular lesion begins with poor repair and results in functional and structural lesions, with vasoreactivity alteration, greater inflammation, altered endothelial permeability, and capillary rarefaction [227]. In preclinical models, an imbalance between EC destruction and regeneration contributes to the transition to chronic vascular lesions [228]. ECs exhibit an expression of caspase-3, an abnormal cytoskeleton, and a disruption to adherens junctions, with increased vessel permeability and apoptosis [229,230,231]. Although dialogue between ECs and pericytes allows for the remodeling and repair of injured vessels [232,233], the loss of pericytes provokes permanent EC damage in peritubular capillaries and is associated with tubular injury in AKI mice [234]. Furthermore, the expression of angiogenic factors (such as vascular endothelial growth factor (VEGF)) are markedly lower [235,236] after kidney injury and inhibited EC proliferation [237,238], whereas the anti-angiogenic factors (such as endostatin) are overexpressed [239,240]. The angiogenic factor silent information regulator two protein (Sirtuin)-1 protects against capillary rarefaction by blocking premature EC senescence secondary to p53 and Notch1 signaling downregulation [241,242,243]. Both ECs and pericytes are able to differentiate into myofibroblasts that also promote kidney fibrosis and progression to CKD [100,244]. Overall, all these factors promote the AKI-to-CKD transition.

#### 3.3.2. Role of UTs in Endothelial Dysfunction during the AKI-to-CKD Transition

UTs (especially IS and pCS) are known to have harmful effects on the vascular wall during CKD (Table 2). In clinical studies, IS and pCS have been linked to cardiovascular disease and mortality [245,246,247]. In preclinical studies, IS exerted direct toxicity on ECs by decreasing cell viability and proliferation and by promoting oxidative stress [83,84,248]. Moreover, IS induced the upregulation of vascular adhesion molecules (such as ICAM-1 and MCP-1) associated with leukocyte extravasation and decreased vascular relaxation [249,250,251]. IS also acts on the calcification process by inducing the expression of osteoblast-specific proteins in VSMC and ECs’ secretion of procalcifying IL-8 [252,253]. Furthermore, IS increased tissue factor production and induced a pro-thrombotic state [254]. Recent studies have revealed a novel antiangiogenic action of IS through the activation of AhR associated with a decrease in EC proliferation [255]. Moreover, plasma angiopoietin-1 concentration increased significantly after hemodiafiltration, a technique used to purify UTs in the dialysate of CKD patients [256]. In the kidney vasculature specifically, IS also exerts toxicity on arterioles obstructed by ECs. After 8 weeks of intravenous IS administration, larger arteries presented reduplication of elastic lamina [69]. Like IS, pCS is associated with cardiovascular events in CKD patients [257,258,259]. Several preclinical studies have investigated pCS’s pathological effects on vascular cells. pCS is responsible for the release of microparticles and an increase in endothelial wall permeability [260]. Furthermore, pCS promotes atherogenesis and plaque instability via its action on leukocyte recruitment and on VSMC migration and proliferation [261,262].

There are now some preclinical data on the pathological vascular role of UTs during AKI. Short-term exposure of ECs to increasing concentrations of IS induced ROS production and ICAM-1 expression and reduced cell viability and NO bioavailability [84,248,249]. Furthermore, IS inhibits EC regeneration via its toxic effect on endothelial progenitor cells (EPCs), with a decrease in their proliferation and an increase in their senescence. In another preclinical study of cultured human EPCs, VEGF expression (usually induced by HIF-1α and IL-10/STAT3 pathways) was suppressed by IS pretreatment [263]. In an animal model of AKI, IS induced a pro-oxidant state with the downregulation of endothelial nitric oxide synthase (eNOs) expression in kidney arteries after I/R and a decrease in EPC egress from the bone marrow [264]. IS also upregulated EC expression of E-selectin in kidney via IL-1β activation, associated with ROS overproduction and greater endothelial monocyte adhesion [265]. In the same manner, ex vivo aorta exposure to IS induced a reduction in vasorelaxation [266,267]. A few studies have demonstrated the harmful action of pCS during AKI. pCS induced ROS production by ECs and VSMCs after only 30 min of exposure in vitro [85]. This effect was associated with vascular leukocyte adhesion and pathological constriction of the aorta in vivo. It was also responsible for an alteration in vascular permeability [268]. Given IS’s ability to block angiogenesis during AKI, this UT probably contributes to the AKI-to-CKD transition. Moreover, IS is able to promote pro-inflammatory cytokines (such as IL-1β) that block the expression of angiogenic factors [235,236,265]. Furthermore, IS is harmful to the EPCs involved in vascular repair [269]. Notably, through its action on vascular wall permeability, pCS might contribute to the AKI-to-CKD transition [260,268].

**Table 2:** Mechanisms of UT toxicity involved in the AKI-to-CKD transition in the endothelial compartment.

UTs	Mechanisms Underlying the AKI-to-CKD Transition	Endothelium		
		Models	Main Results	References
IS	Oxidative stress	In vitro	- ↑ ICAM-1, ↑ MCP-1, ↑ NF-κB, ↑ ROS, ↑ E-selectin, ↑ IL-1β - NADPH oxidase inhibitors decreased IS-induced oxidative stress - ↓ Hypoxia-induced migration and tube formation - ↓ Vasorelaxation	[84,248,249,263,265,266,267]
		In vivo	- ↓ eNOs - AST-120 ↑ neovascularization	[263,264]
pCS	Oxidative stress Inflammation	In vivo	- ↑ ROS	[85]
		In vivo	- ↑ Leukocyte adhesion, ↑ vascular permeability	[268]

Abbreviations: eNOs, endothelial nitric oxide synthase; ICAM-1, intercellular adhesion molecule-1; IL-1β, interleukin 1β; IS, indoxyl sulfate; MCP-1, monocyte chemoattractant protein 1; NF-κB, nuclear factor-κ B; pCS, para-cresyl sulfate; ROS, reactive oxygen species; UT, uremic toxin.

### 3.4. Glomerular Injury

#### 3.4.1. Glomerular Injury in the AKI-to-CKD Transition

During AKI and CKD, immune activation includes the recruitment of different inflammatory cells (predominantly neutrophils and monocytes/macrophages), leading to tubular inflammation. Macrophages are also able to promote the injury of other cells [270]. The podocyte is particularly susceptible to injury and is involved in the AKI-to-CKD transition. In a murine model, podocyte lesions were more prominent than tubular lesions 28 days after I/R injury and characterized by excessive foot process fusion, glomerular basement membrane exposure, and downregulation of proteins with an important role in podocyte integrity (especially synaptopodin and nephrin) [271]. In addition, pro-inflammatory cytokines and angiotensin II cause podocyte lesions and loss, which lead to ROS production and Notch activation [272,273,274]. Notch signaling induced podocyte foot process effacement and EMT, which also promote glomerulosclerosis [273,275]. Furthermore, the senescence of glomerular cells (including podocytes and ECs) leads to senescence-associated secretory phenotypes (SASPs) as part of a switch to a permanent senescence process linked to CKD progression [276]. Glomerular parietal epithelial cells are also involved in the AKI-CKD transition via the formation of crescents [277,278] and the development of glomerulosclerosis [279,280].

#### 3.4.2. The Role of UTs in Glomerular Injury during the AKI-to-CKD Transition

During CKD progression, IS also has harmful effects on glomerular function (Table 3). IS induces podocyte injury both in vitro and in vivo. Mice exposed to intravenous IS for 8 weeks presented elevated albuminuria and histological damage (ischemic damage to the glomerular basement membranes and mesangiolysis) [69]. A specific analysis of podocytes also revealed foot process effacement and cytoplasmic vacuoles. Furthermore, the expression of podocyte proteins (such as synaptopodin) was lower, whereas AhR expression was higher. In vitro, mouse podocytes showed an altered actin cytoskeleton and a switch to a pro-inflammatory phenotype. IS also induced oxidative stress (particularly in mesangial cells) with the production of ROS in a dose-dependent manner [281,282]. Moreover, IS activated heat shock protein 90 (HSp90) through the AhR pathway and promoted renal fibroblast activation [108]. In uremic rats orally supplemented with IS, a histological analysis of the kidney demonstrated higher levels of glomerular sclerosis; these were reversed by AST-120, an oral sorbent that reduced the serum IS level [283,284].

Although pCS is known to have specific toxic effects on interstitial and tubular cells, nothing is known about its potential toxic effects on glomerular cells [285]. Furthermore, there are no data on the potential acute glomerular toxicity of UTs. However, we can assume that IS is also involved in the AKI-to-CKD transition through its harmful glomerular effects. Thus, podocyte and mesangial cell alterations observed after IS exposure are known to contribute to features of the AKI-to-CKD transition, such as ROS production, the upregulation of pro-inflammatory cytokines, cell senescence, fibrosis, and the downregulation of proteins required for podocyte integrity (synaptopodin, nephrin, and CD2AP) [69,271,272,276,281].

## 4. Therapies and Future Research Directions

Today, the only effective way of temporarily reducing plasma concentrations of PBUTs and their complications is renal transplantation. Because of their binding to plasma proteins, PBUTs are poorly eliminated by the various dialysis techniques. Carbon chelating agents (AST-120 (Kremezin^®^), sevelamer carbonate (Renvela^®^)) make it possible to limit PBUTs upstream of their accumulation in plasma, for example, by reducing the passage of their amino acid precursors through the intestinal barrier. However, there is no consensus on their use [286,287,288,289], and there is a lack of data on the long-term effects on improving renal function and reducing cardiovascular complications.

During CKD and AKI, the GM modifies its metabolism, favoring proteolytic fermentation, to the detriment of saccharolytic fermentation [93,290]. Several clinical studies in patients with CKD have shown that a high-fiber, low-protein diet reduces plasma concentrations of UTs [291,292,293,294,295]. However, further long-term studies of nutritional intervention are needed to assess the contribution of single or repeated administration of specific foods or nutrients to plasma retention of UTs in patients. SGLT inhibitors also restore the proteolytic/saccharolytic fermentation balance [296,297,298]. Another strategy targeting the absorption phase is the use of prebiotics, probiotics, and postbiotics. Their use is also controversial, with in vivo data showing efficacy [299] and clinical studies showing mixed results [300,301]. A randomized double-blind clinical trial in 29 patients with chronic renal failure showed that administration of enzobiotic therapy (synbiotics with proteolytic enzymes) for 3 months reduced plasma pCS concentrations with an eGFR that remained stable compared with the placebo group [302]. Fecal microbiota transplantation is another avenue worth exploring to limit the endosynthesis of UTs [303,304,305]. However, preclinical studies show contradictory results in terms of the benefit in animals with AKI [306,307]. To our knowledge, there are currently no clinical studies looking at fecal transplantation in the context of renal disease, and a standardization before setting up such studies is needed (pre-treatment with ATB, galenic form, frequency of administration, inter-individual variability, and management of adverse effects). Certain classes of drugs, such as vancomycin, can be used to restore an altered GM [308]. Genetic modification of the Bacteroides strain no longer expressing tryptophanase may decrease the plasma accumulation of UTs [309].

Another approach is to intervene in the hepatic metabolism of UTs by inhibiting the hepatic enzymes that synthesize them. Thus, in a mouse model of acute renal failure, administration of molecules that inhibit the hepatic enzyme SULT1A1 induces nephroprotection against the renal toxicity of IS [310,311]. Similarly, the study of the inhibition of the FMO3 responsible for the activation of trimethylamine to TMAO may be a therapeutic pathway [312].

PBUTs are very poorly eliminated by conventional dialysis methods. In order to optimize PBUT elimination, various alternative techniques are being studied, such as the combination of diffusive and convective techniques in pre- or post-hemodiafiltration [313,314,315]. The use of an adsorbent during hemodialysis (albumin, nanoporous monolith, activated charcoal, highly porous microparticles, graphene nanoplatelets) has also shown interesting results [316,317]. Another method is to use molecules with a higher affinity for albumin than UTs (sodium octaonate, furosemide, ibuprofen) [318,319,320,321]. Finally, it is likely that limiting the deleterious effects of UTs will require a combination of all of these therapies.

## 5. Conclusions

Many studies of preclinical models of AKI and CKD have shown that post-injury cellular defense and adaptation mechanisms induce the progression from AKI to CKD regardless of the injured renal compartment (the tubule, glomerulus, or endothelium). After AKI, the induction of an immune response leads to the secretion of proinflammatory and profibrotic factors. This harmful environment modifies the metabolism of tubular cells, glomerular cells, and ECs, which leads to ROS secretion, organelle stress, induction of NF-κB, cellular senescence, EMT, ECM synthesis, and fibrosis. UTs are involved in the same cellular adaptation mechanisms in each kidney compartment, which ultimately lead to chronic inflammation, tubular fibrosis, and progression to CKD. The various cellular and molecular mechanisms described here are interrelated. For example, ER stress and mitochondrial stress are strongly activated under hypoxia. It is therefore difficult to study each mechanism individually. To date, only one preclinical study has specifically investigated the impact of these UTs on the AKI-to-CKD transition. Further studies are therefore needed to investigate the link between UTs and AKI/CKD in order to determine whether these toxins are risk factors and therapeutic targets for the AKI-to-CKD transition.

## Figures and Tables

**Table 1:** Mechanisms of UT toxicity involved in the AKI-to-CKD transition in the tubulo-interstitial compartment.

UTs	Mechanisms Underlying the AKI-to-CKD Transition	The Tubulo-Interstitial Compartment		
		Models	Main Results	References
IS	EMT, TGF-β	In vitro	Extinction of the epithelial phenotype: alteration of the cubic epithelial aspect, ↓ ZO-1, ↓ E-cadherin Mesenchymal expression: ↑ α-SMA, ↑ TGF-β, ↑ Smad2/3/4 pathway, ↑ Snail, ↑ fibronectin, Inhibition of IS-induced EMT: probenecid ↓ EMT-induced MAPK/ERK pathway	[105,106,109,112,113]
		In vivo	Extinction of the epithelial phenotype: ↓ ZO-1, ↓ E-cadherin, Mesenchymal expression: ↑ TGF-β, ↑ α-SMA, ↑ collagen, ↑ vimentin ↑ Fibrosis Inhibition of UT-induced EMT: - Losartan ↓ UTs, TGF-β and Snail and ↓ fibrosis - AST-120 ↓ IS concentration, ↑ ZO-1, ↓ α-SMA, ↓ fibrosis	[106,107,109,110,111,114,115,116,117]
	Oxidative stress	In vitro	- ↑ ROS, ↑ NF-κB, ↓ Nrf2 - N-acetyl cysteine or NF-κB inhibitors or NADPH oxidase inhibitor → ↓ NF-κB, ↓ NOX4	[112,118,119]
		In vivo	- AST-120 ↓ urine and serum IS concentrations, ↑ kidney oxygenation, ↓ 8-OHdG, ↓ interstitial fibrosis, ↑ Nrf2, ↑ HO-1 and ↑ NQO1	[119,120,121]
	Mitochondria dysfunction	In vitro	- ↓ Glucuronidation, ↓ complex II activity, ↓ electron transport capacity - ↓ Fusion, ↑ fission, ↑ autophagy	[122,123]
		In vivo	- ↓ Nitrogen metabolism, alteration of the inner membrane, ↑ fission, ↑ autophagy	[123]
	ER stress	In vitro	- ↓ GRP78, ↑ CHOP	[124,125]
		In vivo	- AST-120 ↓ CHOP	[125]
	Metabolic reprogramming	In vitro	- ↑ AhR-dependent arachidonic acid pathway-reprogramming monocytes	[126]
		In vivo	- Alteration of metabolic pathways, including tryptophan metabolism	[127]
	Epigenetic alteration Cycle cell arrest Senescence	In vitro	- Methylation of CpG islands of sFRP => Wnt/β-catenin pathway activation - Activation of p53 and p21 - NF-κB inhibitors suppressed IS-induced p53 and p21	[128,129,130]
		In vivo	- Administration of recombinant sFRP5 alleviated IS-induced fibrosis - Hypermethylation of Klotho - AST-120 decreased p65, p53, p21, β-galactosidase activation, TGF-β, and α-SMA - Stat3 siRNA suppressed IS-induced β-galactosidase activation and fibrosis	[128,129,130,131,132]
	Inflammation	In vitro	- ↑ Expression of IL-1β, IL-6, IL-15, IL-6 and IL-15, TGF-β, NF-κB, Smad, Stat, B2m, Bax, and Bcl2 - Indole-3-propionic acid suppressed IS-induced MCP-1 - Correlation between CD14 + CD16+ monocytes and plasma IS concentration in AAA patients - Plasma IS from AAA patients promotes IL-10, PPARγ, TGF-β, TIMP-1, IL-6, CCL2, and COX2	[133,134,135,136]
		In vivo	- ↑ MCP-1 - Indole-3-propionic acid ↓ IS-induced MCP-1 - AST-120 ↓ IS-induced Mac-1	[134,135,137]
	Iron death pathways	In vitro	- ↑ Hepcidin expression through AhR - ↑ Intracellular Ca^2+^ concentration and ceramide concentration in erythrocytes - ↑ Eryptosis and thrombosis	[138,139,140,141]
		In vivo	- AST-120 ↓ IS-induced hepcidin expression	[138]
IAA	Iron death pathways	In vitro	- ↑ Eryptosis and thrombosis	[141]
pCS	Inflammation	In vitro	- ↑ Expression of IL-1β, IL-6, IL-15, IL-6 and IL-15, TGF-β, NF-κB, Smad, Stat, B2m, Bax, and Bcl2	[133,134]
Me2PY Me4PY	Oxidative stress	Clinical study	- Correlation between Me2PY or Me4PY and the oxidative stress marker GSH	[142]
CMPF	Mitochondrial dysfunction	In vitro	- Hemodialyzed patient serum with CMPF inhibited ADP-stimulated oxidation	[143]
	Iron death pathways	In vitro	- ↓ GSH levels and GPX4, FHC, FLC, and ↑ intracellular iron concentration	[144]
D-serine	Cycle cell arrest	In vitro	- Activation of GCN2 → senescence	[145]
ADMA	TGF-β	In vivo	- ↑ TGF-β, ↑ α-SMA, ↑ collagen, ↑ fibronectin, ↑ fibrosis	[146]

Abbreviations: 8-OHdG, 8-hydroxyguanosine; AAA, abdominal aortic aneurysm; ADMA, asymmetric dimethylarginine; AhR, aryl hydrocarbon receptor; α-SMA, α-smooth muscle actin; B2m, β-2 microglobulin; Bax, Bcl-2–associated X protein; Bcl2, B-cell lymphoma 2; CCL2, chemokine ligand 2; CHOP, C/EBP homologous protein; CMPF, 3-carboxy-4-methyl-5-propyl-2-furanpropionate; COX2, cyclooxygenase 2; EMT, epithelial-mesenchymal transition; ER, endoplasmic reticulum; FHC, ferritin heavy chains; FLC, ferritin light chains; GCN2, general control nonderepressible 2; GPX4, glutathione peroxidase 4; GRP78, glucose-regulated protein 78; GSH, reduced glutathione; HO-1, heme oxygenase-1; IAA, indole-3-acetic acid; IL, interleukin; IS, indoxyl sulfate; Mac-1, macrophage-1 antigen; MCP-1, monocyte chemoattractant protein 1; Me2PY, N-methyl-2-pyridone-5-carboxamide; Me4PY, N-methyl-4-pyridone-5-carboxamide; NF-κB, nuclear factor-κ B; NOX4, NADPH oxidase 4; NQO1, NAD(P)H quinone dehydrogenase 1; Nrf2, nuclear factor (erythroid-derived 2)-like 2; pCS, para-cresyl sulfate; PPARγ, peroxisome proliferator-activated receptor-γ; ROS, reactive oxygen species; sFRP, secreted frizzled-related protein; TGF-β, transforming growth factor β; TIMP-1, tissue inhibitor of metalloproteinase 1; UT, uremic toxin; ZO-1, zonula occludens-1.

**Table 3:** Mechanisms of UT toxicity involved in the AKI-to-CKD transition in the glomerular compartment.

UTs	Mechanism Underlying the AKI-to-CKD Transition	Endothelium		
		Models	Main Results	References
IS	Podocyte loss and senescence	In vitro	- ↑ AhR, ↑ vimentin - Podocyte effacement, with ↓ in *Actn4*, *Cd2ap*, *Myh9*, *Nphs1*, *Nphs2*, *Podxl*, *Synpo*, and *Wt1* mRNA - ↑ reduction rate, ↑ ROS, ↑ SOD sensitivity in mesangial cells	[69,281]
		In vivo	- ↑ AhR, ↑ vimentin - Podocyte effacement, with ↓ in *Actn4*, *Cd2ap*, *Myh9*, *Nphs1*, *Nphs2*, *Podxl*, *Synpo*, and *Wt1* mRNA - AST-120 ↓ glomerular sclerosis	[69,283,284]

Abbreviations: AhR, aryl hydrocarbon receptor; IS, indoxyl sulfate; ROS, reactive oxygen species; SOD, superoxyde dismutase; UT, uremic toxin.
